# Polyetheretherketone (PEEK) as a Biomaterial: An Overview

**DOI:** 10.7759/cureus.44307

**Published:** 2023-08-29

**Authors:** Shambhavi Moharil, Amit Reche, Khushboo Durge

**Affiliations:** 1 Public Health Dentistry, Sharad Pawar Dental College, Datta Meghe Institute of Higher Education and Research, Wardha, IND; 2 Periodontics, Sharad Pawar Dental College, Datta Meghe Institute of Higher Education and Research, Wardha, IND

**Keywords:** biomaterial, peek modification, restoration, bone resorption, peek implants, dental prosthesis, peek

## Abstract

Polyetheretherketone (PEEK) is a very powerful biomaterial that is increasingly used in dentistry. It has superior properties, which make it desirable in implantology. The applications of PEEK include finger prosthesis, RPD and FPD framework, and dental implants. Changes in the production of polyketone-based materials have been made to ensure consistent production of polymers for medical applications. PEEK is a high-performance semicrystalline material that has physical properties such as high resilience and strength. It is a tooth-colored material, making it desirable for its aesthetic appearance. Traditional manufacturing methods like injection molding, extrusion, and compression molding are used for PEEK. Despite the high price of the polymer, the additional value that PEEK materials bring by offering the possibility of manufacturing parts include lightweight, strength or toughness and able to survive longer in harsh environments. PEEK has trauma or shock cancelling abilities, fracture resisting abilities, stress distributing ability, osseointegrating abilities, With such great qualities PEEK has an increased demand in the market, and this biomaterial never failes to surprise with its amazing success rate. Even in dentistry PEEK has a wide range of applications which includes, as a dental implants biomaterial, prosthetic material, abutment material, post and core material, crowns, removable partial denture framework. With such a huge range of applications PEEK is said to have been providing an all in one package for dentistry. PEEK biomaterial shows great compatibility with bioactive materials which has proven to be of great help to mankind as not only it is involved in life sciences but also in automotives and aerodynamics as well. The main motto of this review is to know the qualities and the properties of PEEK as a capable implant prosthesis for its application focusing on dental implants. This review tells us about the challenges faced when using this material and benefits and advantages of this biomaterial.

## Introduction and background

Polyetheretherketone (PEEK) is a synthetic material with a wide range of applications due to its remarkable properties that benefit mankind. These applications include its use as a biomaterial for dental implants, prosthetic materials, abutment materials, post and core materials, crowns, and removable partial denture frameworks. With such a broad range of applications, PEEK is considered an all-in-one package for dentistry. Reducing marginal bone loss during functional loading is crucial for the success of dental implants.

This review focuses primarily on understanding the qualities and properties of PEEK as a capable material for implant prostheses, specifically in the context of dental implants. PEEK is a semi-crystalline material with a significantly high melting point [1]. As a thermoplastic polymer, it has excellent thermal and mechanical properties, making it a desirable biomaterial for various prostheses. PEEK also has exceptional heat tolerance and chemical resistance, along with a stable structure that enhances its strength. Its low elastic modulus, similar to that of human bone, helps mitigate issues arising from stress and tension.

The dental industry is constantly in search of improved materials to address the limitations of current options. PEEK is a high-performance material known for its high resilience and strength. Its tooth-colored appearance further enhances its appeal for aesthetic applications. For a variety of fixed and removable dental prostheses created with CAD-CAM technology, the use of PEEK has been recommended. It has also been suggested for provisional restorations, implant abutments, custom healing abutments, intra-radicular posts, and occlusal splints. However, only a limited number of clinical studies support these claims [2]. Ma et al. have described PEEK as having an aromatic molecular backbone, featuring a combination of ketone and ether functional groups between the aryl rings [3]. Despite its excellent qualities, there are some disadvantages to current materials, such as the loss of natural teeth and bulkiness, which can affect prosthesis retention and patient satisfaction. PEEK biomaterials demonstrate excellent compatibility with bioactive materials. This has proven beneficial not only in life sciences but also in the automotive and aerospace industries.

## Review

Key characteristics of PEEK

PEEK is known to maintain its properties over a wide temperature range. This biomaterial also has limited inherent osteoconductive properties. Because of its excellent long-term creep, fatigue, stiffness, and strength characteristics, PEEK is employed in fields such as orthodontics, prosthodontics, and orthopedic surgeries. Literature suggests that PEEK can be considered a secondary option to titanium in orthopedics and trauma care [3,4]. Moreover, PEEK can replace titanium abutments and metallic restorations to minimize the risk of neck resorption [3,4]. It also exhibits a low coefficient of friction, coupled with high abrasion and cut-through resistance. PEEK can withstand a wide array of chemicals and harsh conditions at elevated temperatures, making it highly advantageous in both dental and medical applications. One of its significant benefits is low permeability, along with resistance to steam, water, and brine, and minimal moisture absorption. Its electrical properties are stable across a wide frequency and temperature range, and it is naturally flame-retardant. PEEK is inherently pure, producing less outgassing and particle contamination.

Several studies have demonstrated PEEK's biocompatibility. Its physical and chemical properties make it suitable for use in the oral cavity. Technological advancements have shown that PEEK can be manipulated either conventionally or through CAD/CAM processes. Its stress-absorbing and fracture-resistant properties make it even more unique and desirable. Because PEEK's modulus of elasticity is similar to that of bone, it is readily accepted as an implant material. Many reports have highlighted cases of metal toxicity and allergic reactions; with its low reactivity or sensitivity and low solubility, PEEK can serve as a substitute for metallic alloys. Orthodontists have also reported using PEEK-fabricated wires because they exert optimal orthodontic force. Due to these excellent properties, PEEK is considered a high-performance polymer. Despite positive reviews concerning its long-term use, further investigation is still needed [5].

Table 1 shows various categories of polymers and illustrates the position of PEEK under "high-performance polymers".

**Table 1:** PEEK: High-Performance Polymer PEI: polyetherimide, PES: polyethersulfone, PEEK: polyetheretherketone, PEKK: polyetherketoneketone, ABS: acrylonitrile butadiene styrene, PC: polycarbonates, PA: polyamide, PET: polyethylene terephthalate, PMMA: polymethyl methacrylate, PVC: polyvinyl chloride, HDPE: high-density polyethylene, LDPE: low-density polyethylene.

High-Performance Polymers (Amorphous)	High-Performance Polymers (Crystalline)	Engineering Polymers (Amorphous)	Engineering Polymers (Crystalline)	Commodity Polymers (Amorphous)	Commodity Polymers (Crystalline)
PEI	PEEK	ABS	PA	PMMA	HDPE
PES	PEKK	PC	PET	PVC	LDPE

Restrictions or drawbacks of PEEK

While there are numerous advantages to this biomaterial, certain drawbacks limit its use. PEEK is a costly material, making it unaffordable for many; thus, it is only suitable for highly demanding applications. Due to the high temperatures required for its processing, specialized machinery is needed, and the process itself is considered technique-sensitive. Certain chemicals, such as concentrated sulfuric, nitric, and chromic acids, as well as sodium and halogens, can damage PEEK. It also has low resistance to UV light. While PEEK crowns have been reported to reduce stress on the abutment, PEEK abutments increase stress on the crown. This elevated stress can lead to poorly fitted screws and even fractures of both the screw and crown [4]. Furthermore, despite its generally low sensitivity and reactivity, a few cases of PEEK toxicity have been reported [5].

Processing requirements for PEEK polymers

PEEK can be processed using traditional methods such as compression molding, extrusion, and injection molding, among others. However, the crystallinity and, consequently, the mechanical properties of PEEK can be influenced by the processing conditions employed. PEEK can be processed in the temperature range of 370 to 420°C as a linear thermoplastic. Importantly, no corrosive gases are released during its production. Due to the absence of periodontal ligaments, proprioception at the implant-bone interface is decreased, which can occasionally result in excessive stress on the restoration and fractures in the porcelain. Occlusal forces affect the prosthesis, the implant, and the surrounding bone. Factors such as the direction and magnitude of the load, the material of the prosthetic, the design of the prosthesis, the material of the implant, the design of the implant, the number of implants, and the interface mechanism between the bone and implant, as well as the type of bone, collectively influence the stress load on the bone. Such conditions can lead to bone resorption [4].

Conventional implants designed to mend bony defects have traditionally been composed of titanium and its alloys, boasting commendable stability and a range of desirable properties. Despite these advantages, such materials come with several shortcomings, such as radiopacity, osteolysis, allergic reactions like hypersensitivity, and the release of metal ions [6,7]. Specifically, titanium possesses an elastic modulus exceeding 100 GPa, providing a buffer against stress and minimizing resorption of adjacent bone [8]. To circumvent these limitations, PEEK and PEEK-based materials have emerged as viable alternatives to titanium and its alloys, with the aim of mitigating adverse post-implantation complications [9]. However, when compared to implant-supported fixed dental prostheses, traditional complete dentures face the challenge of needing to replace the premolars and molars over time, owing to wear and tear of the artificial teeth after implant placement [1].

When modified with various fillers, PEEK can serve as an effective material for dental crowns and bridges. In its unmodified form, however, PEEK is bioinert and inherently hydrophobic. To reduce the contact angle and impart hydrophilic properties to the unaltered PEEK, the incorporation of fillers is necessary [1]. Veneering PEEK over a titanium prosthesis enhances stress resistance, particularly at the junction between the implant and the bone [10].

Enamel wear or tooth wear due to dental prostheses and restorations is a common occurrence. It is important that the wear induced by any dental restoration does not exceed physiological wear levels. Therefore, the appropriate material should be selected; one that provides stability and strength while closely matching the hardness of enamel, yet without causing excessive wear to the enamel structure [1].

PEEK has been successfully used both as a prosthesis material and for manufacturing dental implants. Recently, PEEK has found various applications in dentistry, owing to its fracture resistance, shock-absorbing capabilities, and enhanced stress reloading properties. PEEK is especially prevalent in endodontics. Its shock-absorbing quality, resistance to fracture, and strength make it an attractive option for the post-core system. One of the notable qualities of PEEK is its stress-distributing ability, which prevents both restoration breakage and root fracture. By diverting and distributing stress, PEEK reduces the forces transferred to both the restoration and root, making it an extensively used material for endocrowns in post-endo treatments [1].

Both in vitro and in vivo studies have indicated that nano-structured PEEK surfaces created by etching with sulfuric acid and rinsing with distilled water facilitate faster osseointegration compared to unmodified PEEK [10]. Additionally, an RCT by Koutouzis et al. revealed a noticeable difference between titanium and PEEK abutments [11].

Chemical structure

Many studies have shown that PEEK can be used as an alternative to titanium in medical and dental applications. MA et al. have suggested that PEEK has an aromatic molecular backbone with a combination of ketone and ether functional groups between the aryl rings [3]. Compared to materials like cortical bone, titanium, and ceramics, PEEK has an extremely low elastic modulus. This property has garnered PEEK numerous applications in both the dental and medical fields [12].

**Figure 1:**
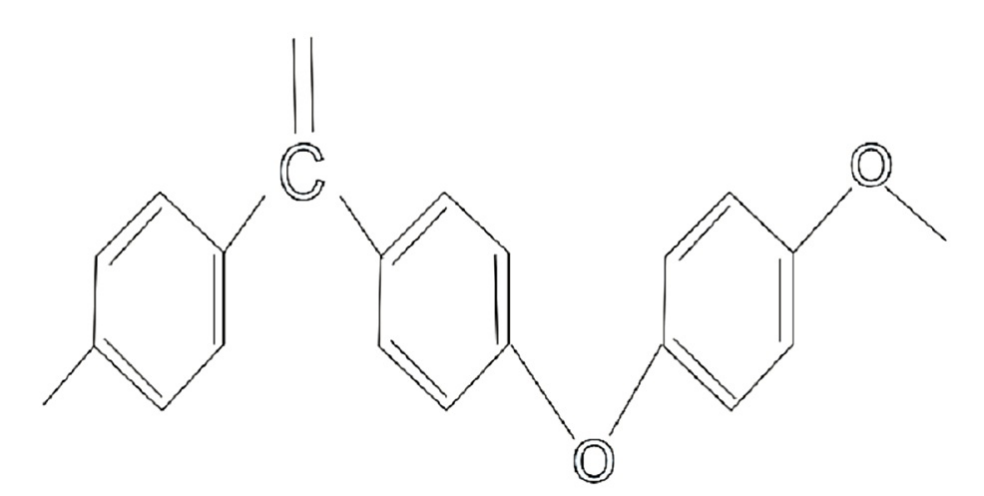
Chemical Structure of PEEK PEEK: polyetheretherketone.

When PEEK is not modified, it exhibits different properties compared to modified PEEK. For example, unmodified PEEK is hydrophobic, while modified PEEK can have increased hydrophilicity, which in turn leads to increased cellular proliferation [13-16]. Figure 1 shows the chemical structure of PEEK, which is an aromatic, semi-crystalline linear thermoplastic polymer.

Use of PEEK in dentistry

Implants

With such excellent advantages, properties, and high biocompatibility, PEEK has become a material of choice in dentistry for various procedures. In implantology, metallic implant materials such as titanium and its alloys have been used, but these also have drawbacks, including disintegration under radiation, sensitivity reactions, allergic reactions, bone loss, and, ultimately, implant failure. Being metallic, titanium and its alloys show several failures; however, a non-metallic material with similar properties, such as PEEK, can overcome these problems [17].

Orthodontic Wires

Orthodontic wires play a significant role in treatment and require high strength. While materials like titanium-molybdenum and nickel-titanium have been used, aesthetic concerns exist. PEEK can be used for aesthetic orthodontic treatment and also offers greater strength [18].

Abutments

Implant-based treatment procedures are serious matters; the materials used must meet all requirements. Titanium, nickel, zirconia, ceramic, and gold are commonly used as abutments. However, they have major disadvantages, including corrosion, osteointegration issues, and allergic reactions. PEEK, being a biomaterial, is considered when such problems need to be avoided. Moreover, abutments made of PEEK can be easily removed if necessary. Due to its low modulus of elasticity, PEEK abutments can withstand greater stresses and forces [19].

Prosthesis: Fixed and Removable

In an implant-supported prosthesis, the crown part forming the upper structure is often metallic but can be corrosive and cause allergies. PEEK, with its higher biocompatibility, doesn't cause allergic reactions. Additionally, PEEK will not exhibit a galvanic reaction when different metals are present in the oral cavity. It also has greater resistance to breakage with CAD-CAM technology and higher wear resistance, making it a competitor to various metallic alloys. Being a non-metallic material, PEEK does not impart a metallic taste or odor and has a low allergy rate. It is easy to polish, resulting in less plaque retention. As an aesthetic material, PEEK can be used to produce metal braces and hooks for orthodontic treatment. It can also serve as an alternative material for removable partial prostheses due to its strength and other properties [20]. Levels of bone matrix proteins in machined, unfilled PEEK were found to be similar to those in raw titanium. Mineralization was possible to some extent in all material variations [19].

The varying responses observed in this study at different time points indicate that several factors such as the material's composition (unfilled PEEK), exposure to industrial processing, surface roughness, and topography can influence osteoblasts' biological response to PEEK. Despite variations in how human osteoblasts reacted to different PEEK samples, implantable-grade PEEK generally matched rough titanium's ability to generate bone in vitro [21]. PEEK has been extensively used in various surgical domains like spine surgery, orthopedic surgery, and maxillofacial surgery. PEEK forms such as PEEK-LT1, LT2, and LT3 have already been used [22]. The application of PEEK-based materials spans a wide range of medical sectors, including bone and cartilage replacement [22-24].

Utilization of Ultra-High Molecular Weight Polyethylene (UHMWPE) and High-Performance Polymers (HPPs) as Dental Materials

Ultra-High Molecular Weight Polyethylene is a thermoplastic similar to PEEK. Beyond its use in robotics, UHMWPE has also found success in various branches of dentistry. For example, UHMWPE ribbon-reinforced composites are used for retention, as post and core, for splinting purposes, and as abutments [25].

Cranial and Maxillofacial Bone Defects

PEEK has been employed alongside other materials for implantation in cases of maxillofacial bone defects. It exhibits high strength, low rates of post-surgical complications like pain and infection, and high fracture resistance. It also shows high stiffness following surgical intervention. PEEK biomaterials offer greater dimensional stability, particularly for implants placed in the cranium, and are resistant to steam sterilization. All these factors suggest that PEEK is an excellent choice for cranial and maxillofacial implants [26-31].

Restorative Dentistry

An aesthetic restorative material is characterized by translucency and a shade close to natural teeth. Although PEEK has a white-colored surface, shading is required to some extent. PEEK's water-repelling property and low surface energy make it less than ideal for long-lasting adhesive force. However, bond strength can be enhanced by surface etching with sulfuric acid and air abrasion with alumina. Although this process may lead to water retention, etching balances this out by creating a rough surface, which enables stronger bonding with moisture-repelling composites. In the future, PEEK has the potential to succeed as a restorative material [32,33].

**Figure 2:**
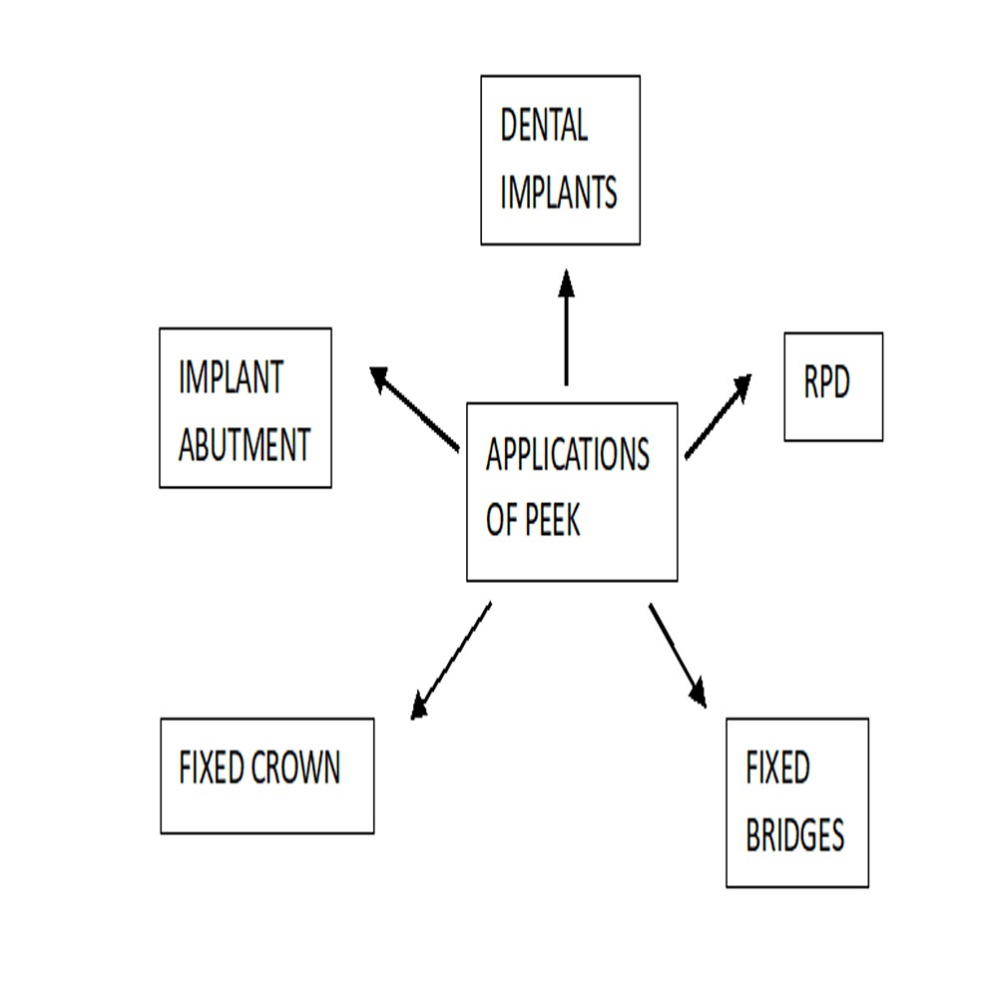
Applications of PEEK PEEK: polyetheretherketone.

Figure 2 illustrates the various uses of PEEK in dentistry, as indicated by the arrows.

## Conclusions

PEEK is a material that has a few great properties and a few drawbacks. Its use after modification yields better results, and it is widely used in the medical as well as dental fields. Specialties such as prosthodontics, periodontics, pedodontics, orthodontics, and orthopaedics readily use this material. There are several uses of PEEK in life science. Despite various setbacks and drawbacks, it still manages to serve mankind. There is no carcinogenicity or mutagenicity associated with the use of this material. PEEK offers an alternative to various other materials such as zirconium and titanium. Since it shows good color stability and appearance, and has an elastic modulus similar to that of cortical bone, it is readily and widely used for dental implants and various other prostheses.

PEEK is used as a temporary abutment since it is more likely to fracture the enamel or not resist masticatory forces compared to titanium and other high-resistant materials. Hence, a PEEK abutment trial is carried out in clinics, and its size, shape, and various other considerations are corrected or modified accordingly in the lab. The patient uses this temporary PEEK abutment for a while, such as a few weeks or even months, but afterward, a definitive permanent prosthesis like an implant becomes mandatory. When a material has disadvantages and drawbacks, it becomes necessary for that material to be modified for its longevity of use and the betterment of mankind. Hence, when PEEK is combined with other materials, like carbon fibers, it becomes a desirable material in dentistry as well as in the medical field. The development of implant biomaterials that exhibit no or minimally harmful effects on host tissues is necessary, given advances in healthcare and the population's longer life expectancy. Although conventional materials like titanium or its alloys are frequently utilized and aid in osseointegration, they have several drawbacks, including the release of metal ions and debris, darkening of the implant area (metallosis), and poor local area stress shielding and visibility.

Different approaches to functionalizing PEEK surfaces and altering the material's structure have been suggested to enhance the material's osteoinductive and antibacterial properties. The use of PEEK-based materials in a wide range of medical sectors includes the replacement of bone and cartilage. PEEK has a bright future in dentistry; whether it be for prosthesis, post and core, crown and bridge, orthodontic treatment, adhesion, restoration, or RPD framework, PEEK biomaterial will likely have a high success rate. In short, the use of PEEK in dentistry will help in resolving various problems, right from aesthetics to osteointegrity.
